# The Success of Serious Games and Gamified Systems in HIV Prevention and Care: Scoping Review

**DOI:** 10.2196/39915

**Published:** 2023-09-05

**Authors:** Waritsara Jitmun, Patison Palee, Noppon Choosri, Tisinee Surapunt

**Affiliations:** 1 College of Arts, Media, and Technology Chiang Mai University Chiang Mai Thailand; 2 Data Analytics and Knowledge Synthesis for Healthcare (DAKSH) Research Group College of Arts, Media, and Technology Chiang Mai University Chiang Mai Thailand

**Keywords:** HIV, serious game, gamification, public health, primary health care, patient care, behavioral health

## Abstract

**Background:**

AIDS, which is caused by HIV, has long been one of the most significant global public health issues. Since the beginning of the HIV epidemic, various types of nonelectronic communication tools have been commonly used in HIV/AIDS prevention and care, but studies that apply the potential of electronic games are still limited.

**Objective:**

We aimed to identify, compare, and describe serious games and gamified systems currently used in HIV/AIDS prevention and care that were studied over a specific period of time.

**Methods:**

A scoping review was conducted into serious games and gamified systems used in HIV prevention and care in various well-known digital libraries from January 2010 to July 2021.

**Results:**

After identifying research papers and completing the article selection process, 49 of the 496 publications met the inclusion criteria and were examined. A total of 32 articles described 22 different serious games, while 17 articles described 13 gamified systems for HIV prevention and care.

**Conclusions:**

Most of the studies described in the publications were conducted in the United States, while only a few studies were performed in sub-Saharan African countries, which have the highest global HIV/AIDS infection rates. Regarding the development platform, the vast majority of HIV/AIDS gaming systems were typically deployed on mobile devices. This study demonstrates the effectiveness of using serious games and gamified systems. Both can improve the efficacy of HIV/AIDS prevention strategies, particularly those that encourage behavior change.

## Introduction

HIV can cause a chronic infection that leads to disease. Without appropriate medical care, many people with HIV develop immunodeficiency syndrome within 10 years of infection [1]. HIV has long been one of the major global public health issues. In 2018, the estimated numbers of people living with HIV in Africa, Southeast Asia, the Americas, and Europe were 25.7 million, 3.8 million, 3.5 million, and 2.5 million, respectively [2]. Because HIV is a communicable infection that can affect the health status of large groups of people, early detection and treatment for one person can prevent future HIV transmission to other individuals, providing benefits for the overall population [3]. Since the beginning of the HIV epidemic, various international organizations have used different types of communications, such as TV advertisements, billboards, school-based events [4], and technology-based health intervention and prevention tools to educate societies [5-7].

Serious games are digital games created with the purpose of entertaining and achieving at least one additional goal [8]. While digital video games are commonly used in the health care domain for purposes such as training health care professionals [9-14], studies that apply the potential of games as HIV/AIDS intervention and prevention tools are still limited. In contrast with serious games, the term *gamification* is described as “the use of game-design elements in non-game contexts” [15]. Additionally, a gamified system is not a game in itself, while a serious game has elements of a real game and looks and feels like a real game [16]. Gamification interventions can be described by their “game mechanics”—the mechanisms that define how the intervention works [17]. They are a powerful tool to engage employees, customers, and the public that can change behaviors, develop skills, and drive innovation [18]. This approach may be one of the most important social and commercial developments in the next 50 years [19]. The difference between a serious game and a gamified system can be seen in their output formats. If the tool has the clearly declared purpose of enhancing the game players’ performance, it is a serious game. In contrast, if it is an application with embedded game elements, is a gamified system.

The benefits of this scoping review include presenting a compilation of HIV care techniques and best practices. Currently, the available reviews in the HIV literature only address detection, treatment, and prevention [20,21]. However, there is no research that identifies game applications as either serious games or gamified systems. The purpose of this review is to identify, compare, and report on the situation of serious games and gamification for HIV interventions from January 2010 to July 2021. It also provides informative sources for an organization or game developers who intend to use games as communication or education tools for social services for HIV/AIDS prevention and care.

## Methods

The review was conducted using the databases PubMed, Web of Science, and Scopus.

**Figure 1:**
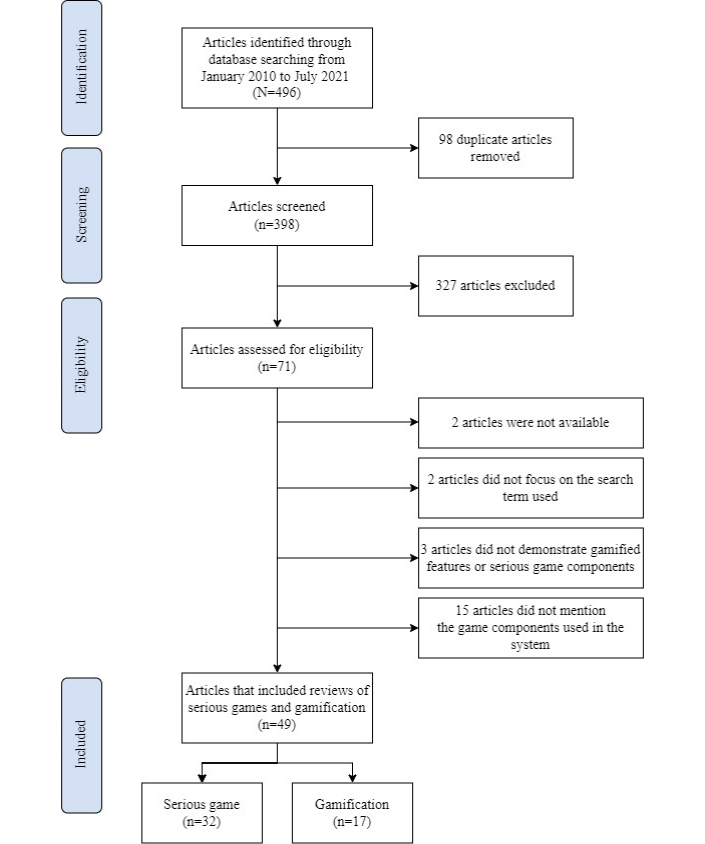
The Preferred Reporting Items for Systematic Reviews and Meta-Analyses (PRISMA) flow diagram.

Although there are various definitions of the terms *serious game* and *gamification*, we decided to use the following search terms: (serious gam* OR videogame* OR video gam* OR gaming OR gamification) AND (HIV). The search results included only articles published in English between January 2010 and July 2021. The procedure is shown in Figure 1.

The criteria that we used to distinguish articles about serious games were as follows: (1) the article included the words (case-insensitive) *serious game* or *serious video game* in its title, keywords, abstract, or main text and (2) the article described a digital game, computer game, or video game created for a purpose related to HIV/AIDS care and prevention. Articles matching either criterion 1, 2, or both were included in this survey.

The criteria that we used to differentiate articles about gamification from those about serious games were as follows: (1) The article included the word (case-insensitive) *gamification* in its title, keywords, abstract, or main text and (2) the article described the use of a gaming approach and game-design components or game elements in a nongame context related to HIV/AIDS care and prevention. Articles matching either criterion 1, 2, or both were included in this survey.

## Results

After the article selection process was conducted, we found that 49 of the 496 publications were eligible for inclusion. Of these, 32 articles were on serious games and 17 articles were on gamified systems for HIV prevention and care. We identified the locations where the research was conducted, what platforms were used for serious games, gamification approaches, the purposes of implementing a serious game or gamification, and outcomes.

### Study Sites

There were 32 serious games and 17 gamified systems described in the 49 articles. The majority of articles (36/49, 74%) mentioned that the study’s scope was limited to the United States. Moreover, 2 of 49 (4%) studies took place in Uganda, 1 (2%) in Swaziland, 2 (4%) in Kenya, 1 (2%) in Tanzania, and 2 (4%) in other sub-Saharan African countries (these were studies that designed and developed digital gamified systems for evaluating students’ perceptions about sexual education). Outside Africa, 1 of the 49 (2%) studies took place in the Philippines, 1 (2%) in Indonesia, 1 (2%) in Ireland, and 2 (4%) in Spain. These results indicate that researchers pay more attention to making use of games and gaming in HIV/AIDS prevention and care in the United States than in other countries.

### Video and Nonvideo Game Systems in HIV Prevention and Care

There were a total of 35 electronic gaming systems developed for the 49 studies. Of these, 22 (63%) were serious games and 13 (37%) were gamified systems.

### Platforms Used for Serious Games and Gamification

A platform is the hardware or electronic system used to run a game [22]. The most pervasive game platforms were the PC (desktop computers and laptops), mobile devices (tablets and smartphones), and game consoles (Nintendo Wii, Sony PlayStation, and Xbox). Mobile devices were the most common platform, with 13 studies using this platform for serious games and 11 for gamified systems. There were 8 serious games and 1 gamified system developed for the PC platform. One study on a serious game and one on a gamified system did not clearly indicate whether they used mobile devices or PCs.

### Purposes of Serious Games and Gamified Systems

Table 1 demonstrates the objectives of serious games and gamified systems. The key objectives of eligible gaming systems were identified. We discovered that serious games for HIV/AIDS prevention and care emphasized education, while gamified systems were aimed at promoting HIV testing and screening. The objectives of each gaming system are elaborated in the “Serious Game and Gamified System Implementation” section of this paper.

**Table 1:** Purposes of serious games and gamified systems used in HIV/AIDS prevention and care.

	Serious games, n	Gamified systems, n
Education on prevention	6	2
Reducing risk behaviors	3	3
Building peer resistance skills	1	0
Promotion of HIV testing and screening	3	4
Encouragement of pre-exposure prophylaxis	2	2
Cognitive rehabilitation	3	0
Physical rehabilitation	1	0
Adherence to and engagement in HIV care	3	3

### Serious Game and Gamified System Implementation

Because the implementation of serious games and gamified systems can differ, this section describes and discusses serious games and gamification separately.

### Serious Games

The implementation of serious games includes 2 important factors: game genre and outcomes (results), as described in the following sections. Table 2 summarizes these factors, as well as the target populations.

**Table 2:** Summary of serious games used for HIV prevention and care.

Title	Genre	Target	Purpose	Results
PlayForward: Elm City Stories [23,24]	Role-playing	Young minority Spanish adolescents	To help players learn about risk behaviors, particularly sexual risk	Significant
Secret of Seven Stones [25]	Adventure	Young adolescents aged 11-14 years	To help prevent unintended pregnancy and sexually transmitted infections	Significant
Socially Optimized Learning in Virtual Environments [26]	Role-playing	Young MSM^a^	To reduce sexual shame among MSM and increase HIV testing rate	Significant
Untitled [27]	Role-playing	Adolescents and young adults	Improve HIV testing and pre-exposure prophylaxis access	Preliminary
BattleViro [28]	Action-oriented adventure	Youth living with HIV	To help increase adherence to antiretroviral treatment	Preliminary
Battle in the Blood [29]	Role-playing	Young populations in the Philippines	To influence behavior determinants of HIV	Preliminary
ViralCombat [30]	Action-oriented adventure	Young MSM	To transmit knowledge, behavior, and skills for increased pre-exposure prophylaxis adherence and HIV prevention	Preliminary
My Future Begins Today [31]	Not identified	Secondary school students in sub-Saharan Africa	Sexual health education	Preliminary
Tumaini [32,33]	Role-playing, simulation	Young Africans in high HIV prevalence settings	To help prevent HIV by delaying first sex and increasing condom use at first sex	Preliminary
Insight (including 5 different games and exercises) [34]	Not identified	Cognitively vulnerable adults with HIV	Improving cognitive and everyday functioning in the target population	Preliminary
Game-based training program [35]	Exergame	Older people living with HIV	To ameliorate some aspects of frailty in individuals with HIV	Preliminary
HealthMpowerment [36]	Not identified	Young MSM&T^b^	To promote HIV-related knowledge and behavior with acceptance of individuals with HIV to prevent the spread of infection	Preliminary
DRAMA-RAMA [37]	Role-playing	Adolescents	To build peer resistance skills to avoid being pressured into risky behaviors such as early sexual behavior	Significant
Brain Powered Games [38]	Not identified	At-risk African children	Cognitive training and rehabilitation	Significant
Captain’s Log (BrainTrain Corporation) [39]	Not identified	Children with HIV	Cognitive training and rehabilitation	Significant

^a^MSM: men who have sex with men.

^b^MSM&T: men who have sex with men and transgender people.

#### Genres of Serious Games

We identified 15 serious games specifically designed for HIV prevention with 4 different genres, as presented in Table 2. There was 1 untitled game. Role-playing was the predominant category of serious games that focused on HIV prevention (n= 6). Among role-playing games, there were 4 educational games, including Play Forward: Elm City, Tumaini, SwaziYolo, and Battle in the Blood [29], and 2 games aiming to reduce risk behaviors, including Socially Optimized Learning in Virtual Environments (SOLVE) and DRAMA-RAMA, the latter of which focused on strengthening peer resistance skills. The second most popular game genre was adventure (n=3), including an educational game called The Secret of Seven Stones (SSS) and an action-oriented adventure game called ViralCombat [30] designed to encourage the use of antiretroviral medications to reduce the risk of acquiring HIV infection. Another game was BattleViro, which encouraged adherence to prescribed antiretroviral treatment (ART). Other genres of serious games among these studies included (1) racing (Fast Car: Travelling Safely around the World, a game that sought to educate adolescents on HIV prevention), (2) a collection of party games (The Test [40], which promoted HIV testing), and (3) an untitled exergame that promoted physical rehabilitation. Another game, My Future Begins Today, which promoted sexual well-being in low-technology settings, had an unidentified genre. [31] Three serious games for cognitive rehabilitation and one collection of games specifically developed to increase adherence to ART also had unidentified genres.

#### Outcomes of Studies on Serious Games

To classify the outcomes of the studies using serious games for HIV intervention and care, we defined 2 types of outcome. The first type was “preliminary results”; these studies focused on proofs of concept for the intervention. Typically, these studies were conducted in a laboratory setting. The second type of outcome was “significant results”; these studies implemented and tested the solution in real-world environments.

#### Serious Games Studies With Preliminary Results

In a pilot study, a theory-based smartphone game titled Tumaini [32] was designed to delay the age of a couple having their first sex and to promote condom use in young Kenyans. The preliminary results indicated that participants who played Tumaini for an average of approximately 27 hours showed significant gains in sexual health–related knowledge and self-efficacy, behavioral intentions for risk-avoidance strategies, and sexual risk communication. This pilot study suggests the need for further research to assess the efficacy of game-based interventions. Another study examined the preliminary effects of Insight [34], a computer program that consists of 5 different games designed to improve cognitive and everyday functioning in older adults living with HIV. The study found that those who performed more poorly at baseline on a useful field of view (UFOV) test that measures visual speed of processing and a test of timed instrumental activities of daily living (TIADL) had more training gains in response to the intervention. In addition, the results revealed that a higher HIV viral load and insufficient medication adherence were predictive of greater TIADL training gains. These results illustrate that middle-aged and older adults with HIV who experience problems with speed of cognition and everyday functioning may benefit from this type of training. Another study evaluated the effectiveness and acceptability of a novel game-based training program (ie, an exergame) [35] aiming to improve various aspects of physical frailty in 10 older persons who were living with HIV. The initial results showed a significant reduction in center of mass sway, a significant increase in gait speed during a motor-cognitive assessment, and a remarkable reduction in reported pain. Other studies examined games aimed at enhancing HIV prevention behaviors and adherence to pre-exposure prophylaxis (PrEP). Viral Combat, a novel and entertaining game or app focused on increasing PrEP adherence and imparting HIV prevention knowledge and skills, was first evaluated through qualitative interviews with 20 young men who have sex with men (MSM). Then, a randomized trial with 60 participants preliminarily tested the game’s effectiveness by evaluating knowledge acquisition, behavior change, and skill improvement after the gaming intervention among young MSM.

Although the Viral Combat study is ongoing, it has returned significant data on changes in motivation to increase PrEP adherence and decrease HIV infection. A mobile game called Battle in the Blood was developed as a role-playing game to influence behavior related to the use of HIV services. During a 12-month study period, the game was installed on 3325 unique devices.

The game received a positive response among casual gamers during playtests. The game’s narrative is a key part of its strategy for delivering HIV knowledge. Another project launched a randomized controlled trial (RCT) to examine the initial effects of an action-oriented adventure game called BattleViro [28], which was developed as a tool to improve adherence to ART, decrease viral load, and increase relevant knowledge in 61 youth living with HIV. The study demonstrated promising results but no significant improvements in HIV knowledge, ART knowledge, or social support. However, those who recently started ART (in the past 3 months) and used BattleViro had a greater decrease in viral load, better ART adherence, and more HIV and ART knowledge than the control group.

One game we identified, in the study by Castel et al [27], never started testing as an intervention by the time that the paper was published, as it was still in a participant recruitment stage. Another game, called My Future Begins Today, was developed for sexual health education and was improved based on user responses in a low-technology setting. The 348 participants (193 boys and 155 girls) were students in secondary school aged 11 to 15 years. The results show that groups using a gamified learning approach outperformed those that used a conventional learning approach. Moreover, a game designed for low-technology settings could be more helpful in delivering sexual health education than formal educational approaches.

Other studies have examined mobile health apps and nonvideo serious games that serve as self-learning tools. These include an app called HealthMpowerment (HMP) [36] and an mHealth app [41] to increase users’ knowledge of HIV and social support pertaining to caring for HIV-positive people and living behaviors of HIV-positive people. The contents were evaluated by a group of young MSM and transgender women in the United States and Indonesia. After testing with the target group, the authors determined that the app-based intervention was easily accessible and could support people with HIV in the long term, resulting in a significant increase in comprehension and knowledge of HIV.

#### Serious Games Studies With Significant Results

We identified 6 serious games with significant results, including PlayForward: Elm City Stories, SOLVE, DRAMA-RAMA (an avatar-based virtual reality game for peer resistance skill building), SSS (a web-based adventure game), and games focusing on cognitive rehabilitation called Captain’s Log and Brain Powered Games.

In a study to evaluate PlayForward [24], a video game that was specifically developed as an HIV prevention tool targeted at HIV risk behaviors (ie, substance use and sex) in young adolescents, the authors showed that after 6 weeks of playing the game, the intervention group had higher knowledge scores than the control group at 6 weeks and at 3 months. Moreover, an analysis of 1,289,903 events in log files of the game revealed that the number of game levels completed was positively correlated with gains in knowledge measured at 6 weeks and at 3 months. These findings demonstrated that PlayForward increased HIV risk–related knowledge among adolescents and that exposure to the video game’s content was highly correlated with knowledge. Another study of the same game [23] analyzed log files from 166 participants in an RCT and showed that higher knowledge scores for substance use at 3- and 6-month follow-ups were related to successfully completing more of the game levels, rather than to total gameplay time.

Another study aimed to investigate the effects of SOLVE [26], a role-playing video game focused on sexual shame reduction among MSM. MSM in the SOLVE intervention reported more shame reduction than MSM in the control condition. Moreover, greater reductions in shame among participants in the SOLVE treatment condition in turn predicted reductions in risky sexual behavior at follow-up. Additionally, SOLVE was the first intervention to significantly reduce shame and risks for MSM from unprotected anal intercourse.

DRAMA-RAMA [37] is an avatar-based video game focusing on peer resistance skill building in teenagers. An RCT was conducted to assess how positively the game was perceived by 45 young Hispanic girls. Separate analyses of covariance showed a significant difference between pre- and posttest scores for peer resistance self-efficacy measured after the game play session, but these findings were not repeated at a 2-month follow-up. The results provided preliminary support for the hypotheses that playing an avatar-based virtual reality technology game would strengthen peer resistance skills and that early adolescent Hispanic girls would have a positive response to this game.

SSS [25] is a web-based adventure game for teenagers aged 11 to 14 years in the United States aimed at education to prevent unwanted pregnancy and sexually transmitted infections. SSS emphasizes educational training to acquire knowledge about sex-related topics and is based on an intervention mapping approach. It can be used at home and is an intergenerational communication channel with parents.

We found 2 studies that examined the efficacy of serious game interventions for cognitive rehabilitation in African children. In the first study, 33 African children with HIV who lived in rural Uganda and were aged between 6 and 12 years played 45-minute sessions of Brain Powered Games several times a week for 2 months. After the intervention, participants demonstrated clinically significant changes on specific Test of Variables of Attention and CogState measures, reflecting processing speed, attention, visual-motor coordination, maze learning, and problem solving. The second study evaluated the neuropsychological and behavioral benefits of Captain’s Log [39], a computer program specially designed for cognitive training and rehabilitation in children living with HIV in Uganda. The 159 participants were randomized to 1 of 3 treatment groups over a 2-month period and were assessed at enrollment, immediately following a 2-month computerized cognitive rehabilitation training (CCRT) course, and 3 months after CCRT completion. The CCRT groups, which used Captain’s Log, had significantly greater gains at the 3-month follow-up compared to a passive control group in an overall mental processing index measured with the Kaufman Assessment Battery for Children, Second Edition (KABC-II); they also showed gains in planning and knowledge. A group that received limited CCRT with the same games rotated randomly from simple to moderate levels of training performed better than a passive control group receiving no training. Moreover, both CCRT arms had significant improvements in CogState Groton maze learning.

### Gamified Systems

Our examination of gamified systems aims to understand 4 important factors: types of gamification approaches, elements of gamification, rewards and incentives used, and effectiveness outcomes, as described in the following sections.

#### Types of Gamification Approaches

In contrast with serious games, gamification is an approach that applies game mechanics in nongame settings. It is used in a broader context and has more specific outcomes. There are typically 3 types of gamification [15]: internal, external, and behavior-change gamification. Internal gamification targets users within an organization, aiming to improve productivity and increase innovation. External gamification is generally driven by marketing objectives to improve relationships between businesses and customers and improves engagement by clients. Finally, the purpose of behavior-change gamification is healthy habit formation among a population. After removing duplicate and conceptual articles describing the initial development phase of gamification projects that lacked a system prototype, we identified 13 different electronic gamified systems in 17 publications. The majority (n=11, 85%) of the surveyed studies used a behavior-change gamification approach and a minority (n=2, 15%) used external gamification. Six mechanics can be distinguished among behavior-change gamification techniques as defined by Werbach and Hunter [15]: challenge, rewards, feedback, resource acquisition, cooperation, and competition. Challenge aims to motivate users to make an effort with content unlocking and requires users’ effort to complete a list of objectives to be fulfilled; rewards are an indicator of players’ achievements; feedback provides information to players about their progress and status during the game through leaderboards or other visual or informational displays; resource acquisition is intended to motivate users to collect and own useful tools; cooperation uses team-building and requires collaboration between players to achieve an objective that is not possible alone; and competition motivates in-game contention between users.

#### Elements of Gamification

We examined the game mechanics that underlie various game components; these directly affect gamification designs [42], as illustrated in Table 3.

The game components used in the surveyed studies comprised avatars, levels, content unlocking, leaderboards, virtual goods, achievements, badges, points, and teams.

**Table 3:** Game mechanics and behavior-change strategies used in behavior-change gamification.

System name	Game mechanics [42]							Behavior change strategies [43]
	Challenge	Rewards	Competition	Cooperation	Resource acquisition	Feedback		
Bijou [44]	No	Yes	No	No	No	Yes	Provide feedback on performance	
Sexual health education programs on the Moodle platform [45]	No	Yes	Yes	No	Yes	Yes	Compare progress; provide feedback on performance	
UBESAFE [46]	No	Yes	Yes	No	No	Yes	Capacity to overcome challenges; provide feedback on performance; reinforcement	
A community-based HIV and sexually transmitted infection testing system [47]	No	No	No	No	No	No	None	
Epic Allies [48,49]	Yes	Yes	Yes	Yes	Yes	Yes	Provide feedback on performance	
AllyQuest [50]	Yes	Yes	No	No	Yes	Yes	Goal setting and social connectivity	
P3 [51]	Yes	Yes	No	No	Yes	Yes	Goal setting; social connectivity; provide feedback on performance	
HealthMpowerment [52,53]	No	Yes	No	No	No	No	Goal setting; social connectivity	
Stick To It [54,55]	No	No	Yes	Yes	Yes	Yes	Compare progress; provide feedback on performance	
Thrive with Me [56]	No	Yes	Yes	No	Yes	Yes	Social connectivity; provide feedback on performance	
Game Plan [57,58]	No	Yes	No	No	No	No	Goal setting	

Avatars are visual representations of players in the game. They are presented to learners as their visual representations and future selves. The avatar component of gamification is used to activate the reward mechanic. In the surveyed studies, we found avatar use in gamified systems including Epic Allies, AllyQuest, P3, HMP, Stick To It, Thrive with Me, Bijou [44], Dot [59], and Game Plan.

Levels show the player’s position at any point during the game to serve as feedback. For example, Besoain et al [46] embedded an experience bar, visualized as medals, that was incremented when users had a significant interaction with the system. In Bijou [44], a completion bar was used to give feedback on the level of completion.

Content unlocking allows a player to access game content or narrative without meeting certain points or specific criteria. The component of content unlocking serves the mechanics of challenge, feedback, and reward.

A leaderboard is a list that shows a ranking of players according to their scores and collections after in-game assessment. The leaderboard allows players to monitor their own ranking and compare it with that of others. The leaderboard component is used to initiate competition and feedback mechanics.

Virtual goods are valuable items that players can purchase during the game in exchange for their points. A representative use of virtual goods was in the game Epic Allies, which used “virtual cards” that players can buy and upgrade using points earned by engaging in other parts of the app. This component is used for the resource acquisition mechanic.

Achievements are rights and rewards given to the player in return for accomplishing an objective; they serve the mechanic of rewards. An example is the gems that are used to signify achievements in Bijou [44].

Badges are commonly used for setting goals, providing explanations about learning activities, identifying players who have shared experiences, and providing users with status. For instance, a badge that shows the level of engagement is given to users in UBESAFE [46]

Points are used to measure success and quantify player progress. In the surveyed studies, users earn points if their responses and in-game actions support healthy behavior and are consistent with HIV prevention measures. Players also earn points in a form of in-app virtual currency in the games AllyQuest and P3. Points can also be earned in the form of scores, as in sexual health education programs with a gamiﬁcation technique [45] and mobile apps. This component serves the mechanics of resource acquisition and feedback.

Teams represent cooperation to achieve common objectives in the game. Participants can recruit team members to achieve in-game challenges in the games Epic Allies and Stick To It. This component serves the mechanic of cooperation.

Additionally, we found that 6 of 7 gamification strategies for health behavior changes determined by behavioral science [43] were discussed in the surveyed articles. The 7 gamification strategies and validated behavior changes were (1) goal setting, (2) increasing capacity to overcome challenges, (3) providing feedback on performance, (4) providing reinforcement, (5) comparing progress, (6) providing social connectivity, and (7) providing fun and playfulness. Only the strategy of providing fun and playfulness was not found to be used in any games. Game mechanics and behavior change strategies that were used in behavior-change gamification in the studies examined here are shown in Table 3.

Moreover, among the surveyed studies, we found one that described a community-based HIV and sexually transmitted infection (STI) testing system [47] that mentioned using gamification techniques, but that article did not clearly explain the game components, game mechanics, or behavior change strategies, except to state that they automated text messages and graphical quizzes. The system appears only to have integrated quizzes into existing systems.

After examination, we found that gamified systems had both educational and advertising purposes using points or scores as the primary element to attract and engage nonpatients who shared similar goals. However, avatar and level elements were commonly used to improve ART adherence in HIV patients.

#### Rewards and Incentives

We found patterns of reward use based on in-game or virtual reward systems [48,50,51] and physical reward systems that allowed exchanging virtual points and in-game currencies into cash prizes or tangible gifts [52-54,56], as illustrated in Table 4.

In addition, there were 3 systems that were classified as using external gamification, that is, social marketing techniques related to a web page’s visitors or use of particular online tools to improve the attitudes of a population toward an HIV testing service and encourage people to physically visit an HIV clinic [60,61] (Table 5).

**Table 4:** Behavior-change gamification, game mechanics, and reward use.

System name	Reward use
Sexual health education programs on Moodle platform [45]	None
Community-based HIV and sexually transmitted infection testing system [47]	None
Epic Allies [48,49]	Buy and upgrade virtual cards using points
Ally Quest [50]	Earn and redeem in-app currency to unlock new app features
P3 [51]	Earn and redeem in-app currency to unlock new narratives and new app features
HealthMpowerment [52,53]	Built-in rewards can be redeemed for physical prizes from the HealthMpowerment online store
Stick To It [54,55]	In-game points increase chance of winning prizes that cost US $5
Thrive with Me [56]	App use can be exchanged for a weekly prize of a US $25 online gift card
Game Plan [57,58]	None

**Table 5:** External gamification and reward use.

System name	Reward use	Game component	Behavior changes strategies
Testing is Healthy [60]	Points can be converted into real-world prizes (eg, food and drinks)	In-game points	Social connectivity (sharing on social media)
Untitled intervention activities [61]	Points can be redeemed for gifts or exchanged for raffle tickets	In-game points	Social connectivity (making use of social marketing principles and social networking sites)

### Outcomes of Studies of Gamified Systems

#### Gamified Systems Studies With Preliminary Results

We identified examples of studies with preliminary outcomes related to the effectiveness of gamified systems that examined the following apps and systems: Stick To It, AllyQuest, Game Plan, a gamified website (HMP), and UBESAFE.

A pilot study assessing Stick To It determined whether an intervention using gamification was acceptable to young MSM in California and effectively increased repeat HIV screening. The preliminary outcome was that engagement in the intervention was moderate; however, the inclusion of game elements was motivating. Additionally, young MSM who enrolled in a clinic were more strongly engaged than young MSM who registered online. Moreover, the repeat HIV screening rate among the subset of participants who were recruited in the clinic was higher than a comparison group of similar young MSM attending the same clinic the previous year. Another pilot study assessed the feasibility and acceptability of AllyQuest [50], a novel smartphone app designed to improve engagement in HIV care and social support among young MSM living with HIV. A pilot trial conducted with 20 participants showed high levels of app use that were positively correlated to HIV self-management outcomes. There was also a statistically significant relationship between the number of days logged into the app and knowledge and confidence in taking HIV medications. These primary outcomes raised the possibility that AllyQuest could impact long-term HIV ART adherence among HIV-positive young MSM. Moreover, a 4-week pilot trial conducted with young Black MSM and transwomen tested HMP [53], a mobile-optimized intervention that included game-based elements to reduce sexual risk behavior by providing information. In this pilot trial, HMP showed promise for being able to deliver a sufficient intervention dose and maintain exposure and engagement over time sufficient to achieve behavioral change. In this qualitative assessment of 15 participants, the researchers found a correlation between the number of times the intervention site was used and stages of behavioral change. Another study explored the initial effectiveness of Game Plan [58], a tablet-based brief motivational intervention for alcohol use and HIV risk. Over 3 months of follow-up, the participants who used Game Plan reported fewer drinking days, fewer binge drinking days, fewer alcohol problems, and fewer new anal sex partners compared to those in the control group. These initial results suggest that web-based brief motivational interventions could be a promising tool to help MSM reduce HIV-related risk behavior. Additionally, a feasibility evaluation of UBESAFE [46] indicated that the gamified features encouraged users to interact more with the system, by up to 100%.

#### Gamified Systems Studies With Significant Results

There were 3 studies of gamified systems with significant results (Table 6), including a pedagogical framework called Motivation, Attitude, Knowledge, and Engagement (MAKE), a promotional campaign aimed at STI and HIV prevention called Testing is Healthy, and the gamified website HMP.

**Table 6:** Results of studies on gamified systems used in HIV prevention and care.

Title	Target	Game’s purpose	Results
Motivation, Attitude, Knowledge, and Engagement framework [45]	Adolescent students aged 11-15 years	Provide pedagogy for sexual health education/improve sexual health education programs for adolescent students	Significant
AllyQuest [50]	Young MSM^a^; mean age of focus group participants was 23 years	Support engagement in care and medication adherence for HIV-positive young MSM	Preliminary
HealthMpowerment [52,53]	HIV-positive and negative young Black MSM and transwomen aged 18-30 years	Use behavior change and gaming theories to reduce risky sexual behaviors and build community with a mobile phone–optimized intervention	Significant
Stick To It [54,55]	College students (aged 18-24 years)	Increase HIV screening among young MSM	Preliminary
Game Plan [57,58]	MSM aged 18-53 years	Help MSM reduce their HIV risk and alcohol use	Significant
Testing is Healthy [60]	Young adults aged 20-29 years	Use digital gaming as a medium for preventing sexually transmitted infections and HIV	Significant

^a^MSM: men who have sex with men.

An RCT was conducted to investigate the extent to which game-based learning and the gamified MAKE framework [45] could improve sexual health education among 120 young students in Tanzania. The adolescents were divided into groups that received teaching with 1 of 3 methods: game-based learning, gamified MAKE, and the control group, which used a conventional teaching method. The results suggested that the first 2 teaching approaches delivered better learning outcomes than the conventional teaching method and could be used as tools to improve sexual health behavior and increase HIV/AIDS knowledge in young students.

The evaluation of Testing is Healthy [60] used a technology-based platform called Cineplex TimePlay that included gaming elements to engage people who often go to the cinema to answer STI/HIV-related questions. The system also provided a clinic finder service on a website. Although this was the first time this platform was used for sexual health promotion, the campaign received a great response, with 548,410 views and 77,149 plays. Additionally, server-side rendering web analytics showed a significant increase in the clinic finder page’s unique page views between the first and second campaigns. The results demonstrate that this technique could be used to reach a large population at a low cost.

The last study [52] was an RCT that compared the efficacy of the HMP intervention to an information-only control website. The results indicated that the rate of self-reported condomless anal intercourse at 3 months was 32%, suggesting that exposure to an online intervention can reduce the rate of condomless anal intercourse among young Black MSM, at least in the short term.

## Discussion

In order to provide a comprehensive map of the available evidence on HIV prevention and care, we used a scoping review methodology to conduct this review. The literature included in this study adhered to the PRISMA (Preferred Reporting Items for Systematic Reviews and Meta-Analyses) standard, which facilitated the inclusion of extensive information on serious games and gamified systems. Nevertheless, this scoping review was restricted to providing answers to particular issues. Therefore, a future systematic review would enhance and enrich our knowledge.

The results of this scoping review show that 74% (36/49) of study sites, as reported in 16 articles that examined serious games and 10 that examined gamification, were in the United States. A small minority of reviewed studies were performed in sub-Saharan African countries, which have the highest global HIV/AIDS infection rates. Because of the unavailability of electricity and communication facilities, the level of technological solutions in rural areas of Africa is very low [62]. Therefore, researchers need to make the most of their resources by applying other communication tools and methods that focus on users’ behaviors and lifestyles instead of depending on digital technology and gaming.

Moreover, the majority of the serious game and gamified systems used mobile platforms. This confirms that improvements in mobile computing hardware and wireless networking improve outcomes and not only make interventions accessible for users but are also beneficial for game developers, as they allow them to create dynamic and realistic worlds and characters [63].

The major purpose of serious games and gamified systems applied in HIV/AIDS studies is to enhance the effectiveness of HIV/AIDS prevention strategies by reducing sexual risk behaviors and increasing the HIV/AIDS screening rate. The surveyed results are consistent with findings from previous studies [64,65] that indicate that early diagnosis and prevention of HIV infection is more cost-efficient than lifetime HIV treatment. Moreover, 6 of the 16 serious games we identified were in the role-playing genre, of which half were designed for educational prevention and reducing risky behaviors.

For our analysis of game elements in gamified systems, we followed the categories proposed by Werbach and Hunter [15] because there is no universal standard to classify game elements in gamified systems. Their taxonomy of game elements is one of the most widely used taxonomies in research on gamified systems [66-69]. The taxonomy includes 15 game components that can represent specific mechanical forms or can be dynamic, including achievements, avatars, badges, boss fights, collection, combat, content unlocking, giving, leaderboards, levels, points, quests, social graphs, teams, and virtual goods. We then attempted to match the selected studies to this taxonomy to determine the characteristics of gamified system components in the HIV domain. The common patterns we observed in the development of gamified systems for HIV are consequently a guide for future studies.

The majority (11/13, 85%) of the surveyed approaches were classified as using behavior-change gamification, which is a technique that seeks to encourage healthy sexual habits in a given population. Some fundamental game components are embedded in the kinds of systems we reviewed here, such as points, badges, leveling, and rewards. However, some systems claimed to incorporate gamification despite the fact that they only used graphical quizzes and had no other defined game elements or strategies. This reflects the complexity of the gamification concept, as well as its application; both the concept and its application are in the introductory stages in the domain of HIV/AIDS prevention and care. Our findings also show patterns of reward use, including in-game rewards and physical prizes, that have the same behavioral change objectives for HIV/AIDS care and prevention.

The in-depth analysis of the effects of game mechanics and behavioral change strategies used in behavior-change gamification shown in Table 3 reveals that the first-ranked, most frequently used game mechanic was rewards. Kocadere and Çağlar [42] indicate that rewards serve as a gauge of a player’s performance; the components of the reward mechanic are badges, achievements, avatars, and content unlocking. Moreover, rewards can be used for players to progress and to create player emotions, including apprehension, excitement, grief, and happiness with dynamic use of constraints, emotions, progression, and relationships [15].

Feedback was the second-ranked mechanic. Feedback is data presented to the player regarding their performance in the game. The feedback mechanic serves the dynamics of progression and emotions. Feedback can be both formative (badges, levels, leaderboards, content unlocking) and summative (points, badges, leaderboards, levels) [42]. The reason that rewards and feedback are used in behavior-change gamification is that player progress and emotions are largely influenced by the rewards and feedback they receive. Therefore, if the goal of the gamified system is to improve the behavior of users, a mechanism for providing rewards and feedback should be considered when developing a behavior-change gamification system.

### Conclusion

As the reach of the internet and cell phones improves, their use as health intervention and prevention tools has increasingly been researched to strengthen their impact on behavior and social change in HIV/AIDS prevention and care. However, the study sites of research on serious games and gamified systems mentioned in articles from 2010 to 2021 were mostly in the United States. This contrasts with the small number of studies undertaken in sub-Saharan Africa, which has the highest global HIV/AIDS infection rate. We also discovered that the majority of serious games use mobile platforms. This finding demonstrates that improvement in mobile computing hardware and wireless networking has made them more accessible for users.

The majority (11/13, 85%) of the surveyed studies were classified as using a behavior-change gamification approach. This is a technique that seeks to form healthy habits among the population. Additionally, the results highlight the intricacy of gamification and its applications, which are still in their infancy in the field of HIV/AIDS prevention and care. Furthermore, the survey results show that the most important goal of serious games and gamification is to improve the effectiveness of HIV/AIDS prevention efforts. This is consistent with findings from previous studies that indicate that early prevention and early ART of HIV/AIDS infection are more cost-efficient than lifetime HIV/AIDS treatment. This study identifies, compares, and describes serious games and gamified systems currently used in HIV/AIDS prevention and care. The study results reveal the situation in the studied period pertaining to common popular study sites, game platforms, and gamification approaches among serious games and gamified systems used in HIVAIDS prevention and care. The contributions of this review should provide a resource of information for organizations, game developers, and other parties who intend to use games as communication or education tools for HIV/AIDS.

## Appendix

Multimedia Appendix 1 Preferred Reporting Items for Systematic reviews and Meta-Analyses extension for Scoping Reviews (PRISMA-ScR) checklist.

## Abbreviations

ART: antiretroviral treatment
CCRT: computerized cognitive rehabilitation training
HMP: HealthMpowerment
MAKE: Motivation, Attitude, Knowledge, and Engagement
MSM: men who have sex with men
PrEP: pre-exposure prophylaxis
PRISMA: Preferred Reporting Items for Systematic Reviews and Meta-Analyses
RCT: randomized controlled trial
SOLVE: Socially Optimized Learning in Virtual Environments
SSS: Secret of Seven Stones
STI: sexually transmitted infection
TIADL: timed instrumental activities of daily living
UFOV: useful field of view
